# Rapid taxonomic categorization of short, abundant virus sequences for ecological analyses

**DOI:** 10.1002/ece3.11501

**Published:** 2024-06-17

**Authors:** Anna R. Sjodin, Michael R. Willig, Armando Rodríguez‐Durán, Simon J. Anthony

**Affiliations:** ^1^ Department of Ecology & Evolutionary Biology University of Connecticut Storrs Connecticut USA; ^2^ Center for Environmental Sciences & Engineering and Institute of the Environment University of Connecticut Storrs Connecticut USA; ^3^ Mata de Plátano Field Station Universidad Interamericana de Puerto Rico Bayamon Puerto Rico USA; ^4^ Center for Infection and Immunity Columbia University New York New York USA; ^5^ Department of Pathology, Microbiology, and Immunology UC Davis School of Veterinary Medicine Davis California USA

**Keywords:** bats, Chiroptera, community ecology, herpesviruses, host specificity, operational taxonomic units, viral ecology

## Abstract

Public health concerns about recent viral epidemics have motivated researchers to seek novel ways to understand pathogen infection in native, wildlife hosts. With its deep history of tools and perspectives for understanding the abundance and distribution of organisms, ecology can shed new light on viral infection dynamics. However, datasets allowing deep explorations of viral communities from an ecological perspective are lacking. We sampled 1086 bats from two, adjacent Puerto Rican caves and tested them for infection by herpesviruses, resulting in 3131 short, viral sequences. Using percent identity of nucleotides and a machine learning algorithm (affinity propagation), we categorized herpesviruses into 43 operational taxonomic units (OTUs) to be used in place of species in subsequent ecological analyses. Herpesvirus metacommunities demonstrated long‐tailed rank frequency distributions at all analyzed levels of host organization (i.e., individual, population, and community). Although 13 herpesvirus OTUs were detected in more than one host species, OTUs generally exhibited host specificity by infecting a single core host species at a significantly higher prevalence than in all satellite species combined. We describe the natural history of herpesvirus metacommunities in Puerto Rican bats and suggest that viruses follow the general law that communities comprise few common and many rare species. To guide future efforts in the field of viral ecology, hypotheses are presented regarding mechanisms that contribute to these patterns.

## Graphical abstract

We sampled 1086 bats from two, adjacent Puerto Rican caves and tested them for infection by herpesviruses, resulting in 3131 short, viral sequences. Using percent identity of nucleotides and a machine learning algorithm (affinity propagation), we categorized herpesviruses into 43 operational taxonomic units (OTUs). We describes the natural history of herpesvirus metacommunities in Puerto Rican bats, suggest that viruses follow general laws of community ecology, and guide future efforts in the field of viral ecology by presenting hypotheses regarding mechanisms that contribute to demonstrated patterns.

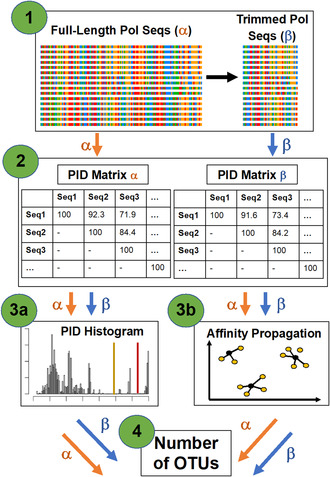

## 1 INTRODUCTION

Ecology has a long history of quantifying the abundance and distribution of organisms in nature, and in recent years has been increasingly focused on the study of viruses (Anthony et al., 2015, 2017; Diaz‐Munoz, 2017; Gao et al., 2022; Holmes, 2022; Jokinen et al., 2023; Malmstrom et al., 2011; Olayemi & Fichet‐Calvet, 2020; Plowright et al., 2015). These efforts have mostly centered on relationships between single host species and single pathogen species, adopting a reductionist approach and studying such systems in isolation. Although this monothetic methodology has been successful in the control of known diseases (e.g., rabies: Benavides et al., 2020; Mshelbwala et al., 2016; Reynolds et al., 2015; Hendra: Eby et al., 2020; Plowright et al., 2008; Plowright et al., 2011; Marburg: Amman et al., 2012; Amman et al., 2020; Towner et al., 2009), it likely falls short of capturing the full complexity and scope of interactions that contribute to ecological patterns. Infected host individuals often contain multiple viruses and transmit them among cohabitating host species. Infection with one virus can affect infection, virulence, or disease emergence of others (Anthony et al., 2015; Seabloom et al., 2015; Telfer et al., 2010). Thus, it is essential to better understand the role of community‐level processes and their potential influence on viral abundance and distribution.

Several challenges exist when integrating community ecology with virology. First, most wildlife viruses are unknown to science (Anthony et al., 2013, 2017; Carlson et al., 2019), and it is difficult to study the ecological processes operating within viral communities without first sorting viruses into species or equivalent units. Additionally, most viruses are rare (i.e., exist at low prevalence; Anthony et al., 2015; Tang et al., 2006; Wacharapluesadee et al., 2010; Winker et al., 2008), and many do not persist in host individuals for long periods of time (Henaux & Samuel, 2011; King et al., 2012; Klenk et al., 2004; Lee et al., 2009; Vetter et al., 2016). Thus, the probability of infection by any one virus at any particular time is low. Furthermore, large datasets of comprehensively sampled host populations are needed to address high levels of uncertainty associated with low viral prevalence. Such datasets are atypical and generally consist of partial, and often only small (e.g., <300 base pairs [bp]) genome sequences. Finally, the process of defining official virus species according to the International Committee on the Taxonomy of Viruses (ICTV) is thorough but also complex and time consuming. Proactively analyzing and identifying ecological patterns for developing public health threats, however, require fast, functional, and biologically relevant viral classification to support scientific study until the more systematic ICTV classifications can transpire. Fortunately, many analyses of community ecology, specifically, do not require identities of species (e.g., the species abundance distribution, empirical cumulative distribution; Fisher et al., 1943; MacArthur, 1957; McGill et al., 2007; Preston, 1960; Raunkiaer, 1909), so alternative classification schemes can be used to show macro‐ecological patterns or compositional trends. For viruses, such schemes include delineating operational taxonomic units (OTUs) using monophyletic groups (Anthony et al., 2015) or pairwise distances (Lauber & Gorbalenya 2012; Maes et al., 2009; Muhire et al., 2014), but these methods rely on partially subjective cutoffs or full‐length sequences to differentiate OTUs. A new, more objective method is needed that utilizes highly abundant and readily available short sequence data and allows timely completion of ecological analyses.

We address some of the challenges associated with studying the ecological processes that shape viral communities using imperfect but comparatively easily gathered data. We use herpesviruses as a model taxon because they generally occur at high prevalence (Cortez et al., 2008; Imbronito et al., 2008; Phalen et al., 2017; Tazikeh et al., 2019) and establish latent, long‐term infections, often for the entirety of the host's life (King et al., 2012). They can also be detected easily using consensus polymerase chain reaction (cPCR; Van Devanter et al., 1996; Wibbelt et al., 2007; Wray et al., 2016) and frequently co‐infect the same host individual (Azab et al., 2019; Elhassan et al., 2016; Seimon et al., 2015).

Herpesviruses are large (~300,000 bp), enveloped, double‐stranded DNA viruses that infect all vertebrate classes and mollusks (Azab et al., 2018; King et al., 2012). They were first detected in bats in 1996 (Tandler, 1996) and have since been reported in many bat species (Holz et al., 2018; Pozo et al., 2016; Razafindratsimandresy et al., 2009; Sasaki et al., 2014; Wada et al., 2018; Wibbelt et al., 2007; Zhang et al., 2012; Zheng et al., 2016). Bats are even suggested to have played a critical role in the evolution and diversification of New World herpesviruses (Escalera‐Zamudio et al., 2016), making them an important host taxon for the study of herpesvirus ecology more broadly. Despite recent advances, much about bat herpesviruses remains unknown, including the diversity of herpesviruses in different bat species, the degree to which bat herpesviruses are host specific, and the factors that contribute to viral sharing between hosts.

In general, herpesviruses are highly host‐specific (King et al., 2012). However, genomic analyses suggest that their evolution is characterized by cross‐species transmissions (i.e., spillover) with subsequent adaptation to novel hosts (i.e., emergence; Azab et al., 2018; Escalera‐Zamudio et al., 2016). Inter‐specific transmission has been documented when close contact occurs between individuals of different species, especially if host species are closely related (e.g., within primates, Weigler, 1992; within testudines, Greenblatt et al., 2005; within ungulates, Russell et al., 2009). Multiple bat species come into close proximity within caves where they roost, presenting opportunities for cross‐species transmission (Simonis & Becker, 2023). Indeed, cross‐species transmission of herpesviruses and host switching are common in bat hosts and have likely been the norm throughout the evolutionary history of these viruses (Azab et al., 2018; Escalera‐Zamudio et al., 2016; Wada et al., 2018; Zheng et al., 2016). Cross‐species transmission is particularly evident within the subfamily Gammaherpesvirinae, as its constituent species show less strict host‐pathogen co‐phylogeny with bat hosts than do viral species within the Alphaherpesvirinae or Betaherpesvirinae (Azab et al., 2018; Escalera‐Zamudio et al., 2016; Wada et al., 2018; Zheng et al., 2016). The ecological or genetic reasons underlying differences in virus sharing between host species are unknown.

The goal of this study was to implement a quantitative, objective approach to determine viral units from a large and relatively easily collected sequence dataset and then use the units to (1) estimate total herpesvirus diversity, (2) describe patterns of community distribution and co‐infection, and (3) examine host‐specificity and spillover for herpesviruses in cave‐dwelling bats of Puerto Rico. Because most viruses are rare (Young & Olival, 2016), and because hyperbolic species abundance distributions (SADs) represent a general ecological law (McGill et al., 2007), we predicted that herpesvirus metacommunities would comprise few common and many rare viruses. Well‐established understanding of strong host‐specificity in herpesviruses, coupled with recent insights highlighting host sharing in bats as an important eco‐evolutionary mechanism shaping herpesvirus diversity more broadly (Azab et al., 2018; Escalera‐Zamudio et al., 2016; Wada et al., 2018; Zheng et al., 2016), suggest that (1) closely related hosts regularly share herpesviruses on an ecological timescale and (2) patterns of specificity (e.g., high infection prevalence in a single host species) develop over longer, evolutionary time periods. We therefore hypothesized that we would see instances of herpesvirus spillover among bat species, but that virus OTUs would show a strong preference for one, primary host species. In these spillover recipients, viruses would exist at a lower prevalence, because the viruses are not as strongly adapted to secondary species. Finally, in concert with common ecological occupancy‐abundance patterns seen in macro‐organisms (Gaston et al., 2000; Gaston & He, 2011; He & Gaston, 2003), we expect that viruses shared across multiple host species will be found at the highest prevalence in the bat community.

## 2 MATERIALS AND METHODS

### 2.1 Sample collection

Field work was conducted at two adjacent caves on the Mata de Plátano Nature Reserve in Arecibo, Puerto Rico (18°24.868′ N, 66°43.531′ W). The 13 bat species on the island are well‐documented and have been studied extensively (Gannon et al., 2005; Gannon & Willig, 1992, 1994, 1997; Rodríguez‐Durán, 1995, 1998, 2009; Rodríguez‐Durán & Soto‐Centeno, 2003). Mata de Plátano Nature Reserve (operated by InterAmerican University, Bayamon, Puerto Rico) is in the north‐central, karst region of the island, an area dominated by a multitude of caves that are suitable for roosting bats.

Culebrones Cave is structurally complex and hot, with temperatures reaching 40°C and relative humidity at 100%. It is home to an estimated 158,000 individual bats representing six species: *Pteronotus quadridens*, *P. portoricensis*, *Mormoops blainvillei*, *Monophyllus redmani*, *Erophylla bombifrons*, and *Brachyphylla cavernarum* (Rodríguez‐Durán et al., 2023). Bats were sampled for 28 nights between June and August 2017. A harp trap was placed at sunset (approximately 18:00) immediately outside of the opening and monitored continually. Larvas Cave is cooler (ambient temperature), smaller, and less structurally complex compared to Culebrones Cave. It hosts a small total number of bats (30–200; ARS personal observation) representing two species, *Artibeus jamaicensis* and *Eptesicus fuscus*. Bats were sampled from Larvas Cave on seven instances between June and August 2017, using two different techniques. After sunset, mist nets were placed along a trail outside of the cave entrance to catch exiting bats and were checked at least every ten minutes. Hand nets were also used to capture bats roosting inside the cave.

Regardless of sampling method, each captured bat was placed into a porous cotton holding bag. Individuals were identified to species following Gannon et al. (2005), and a clean swab (Puritan polyester‐tipped applicator) was used to collect saliva from each bat's mouth. Swabs were placed in individual cryovials containing viral transport medium (Dulbecco's Modified Eagle Medium, 1% Penicillin–Streptomycin, and 1% Amphotericin B) and sent in a dry shipper to Columbia University's Center for Infection and Immunity, where they were stored at −80°C. All methods were approved by the University of Connecticut Institutional Animal Care and Use Committee (IACUC, protocol A15‐032) and the Puerto Rican Departamento de Recursos Naturales y Ambientales (DRNA, permit numbers 2016‐IC‐097 and 2017‐EPE‐006).

### 2.2 Viral screening

Total nucleic acids were extracted from each swab (one swab per bat; 1086 total) using the EasyMag platform (bioMerieux, Inc.), and cDNA was synthesized using SuperScript III first‐strand supermix (Invitrogen). Synthesis of cDNA without DNAse treatment allows for the detection of both viral mRNA and genomic DNA and thus maximizes the likelihood of detection. Nested consensus polymerase chain reaction (cPCR) was performed twice on each sample, targeting a region of the highly conserved catalytic subunit of the DNA polymerase gene (Van Devanter et al., 1996). cPCR is a broadly reactive method that allows detection of both known and novel (i.e., genetic material not yet publicly available) viruses by using degenerate primers targeting viral sequences that are conserved at the level of virus family or genus (Anthony et al., 2013, 2015; Van Devanter et al., 1996). The assay was performed twice to increase chances of herpesvirus detection, thereby partially accounting for imperfect detection associated with this method.

The cPCR protocol was modified slightly from that of Van Devanter (1996) to include the use of the QIAGEN Fast Cycling PCR kit: 95°C for 5 min, then 45 cycles of 96°C for 8 s and 68°C for 12 s, finished with 72°C for 2 min. Primer and dimethyl sulfoxide (DMSO) concentrations were identical to that of Van Devanter et al. (1996). A constructed synthetic plasmid was used as a positive control to confirm effective implementation of the assay and to detect possible contamination. cPCR reactions were resolved on 1% agarose. Products of the expected size (~225 bp) were excised, purified using Ultrafree‐MC Centrifugal Filter (Millipore), and cloned into Strataclone PCR cloning vector. Twelve white colonies were then sequenced using conventional Sanger sequencing. Based on our previous experience, sequencing more than 12 colonies rarely resulted in the detection of additional genetic diversity. However, rare viruses present at very low copy number may have been missed. As rare viruses presumably contribute the least to community assembly processes, we suggest this limitation should not impact our overall findings. All sequences (clones) were cross‐referenced against the GenBank nucleotide database using Blastn to confirm that they were herpesviruses.

### 2.3 OTU classification

**FIGURE 1:**
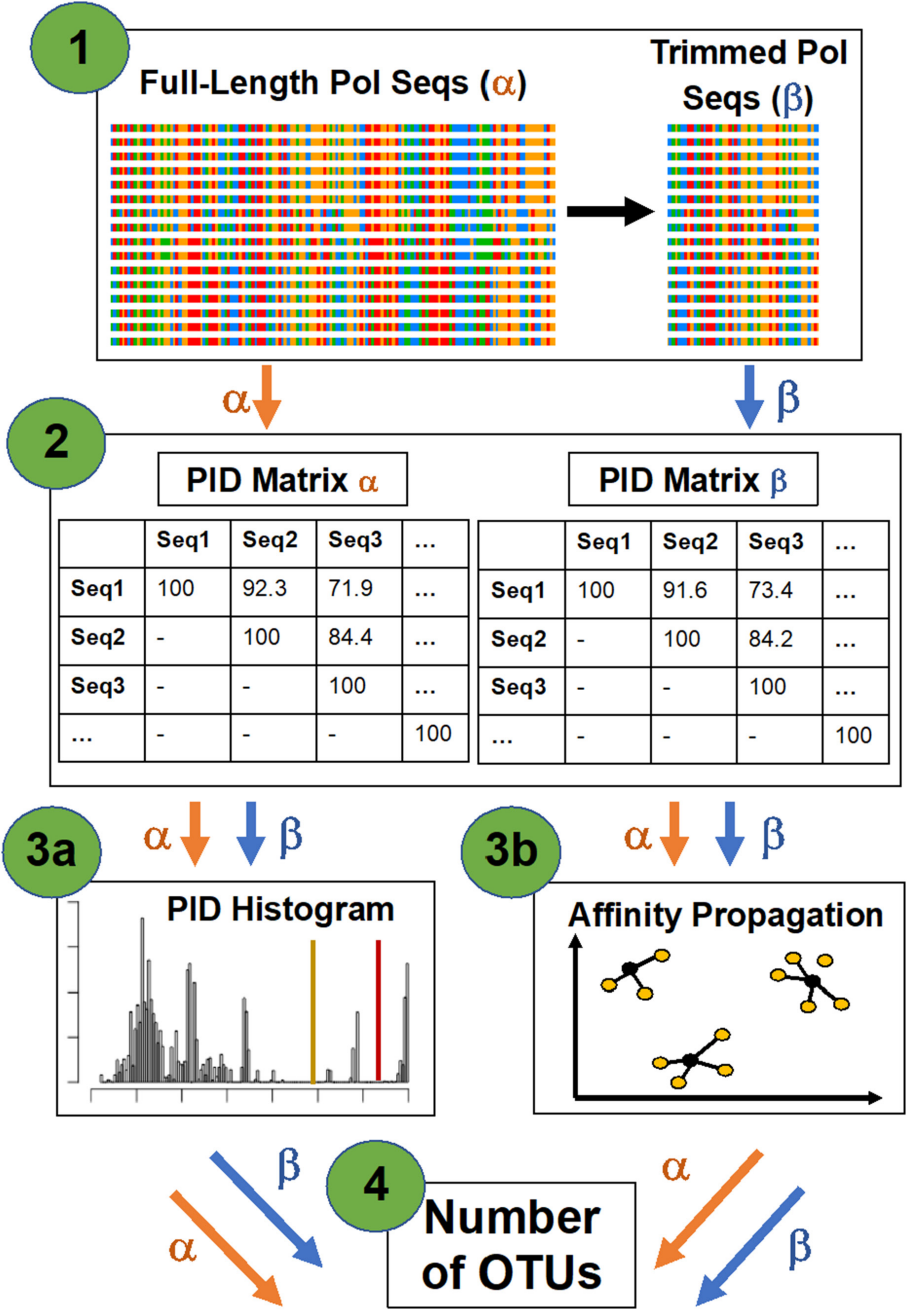
Schematic illustrating the validation methods for determining the number of OTUs. In Step 1, alignments are generated from GenBank sequences representing full‐length polymerase sequences of ratified herpesvirus species (~3000 base pairs). Step 1 also includes trimming GenBank sequences to the same ~178 base pair region as is sequenced for the empirical samples. In Step 2, percent identity (PID) matrices are calculated separately for each alignment. Each PID matrix is then used to create PID histograms (Step 3a) and run affinity propagation (Step 3b). Step 3b (affinity propagation) was repeated 25 times. The results of Steps 3a and 3b were used in tandem to determine the number of OTUs (i.e. virus species) in the alignment (Step 4). “Polymerase” is abbreviated as Pol, and “sequence(s)” is abbreviated as seq(s).

Empirical sequences were aligned using ClustalW, executed in Geneious version 11.0.5 (https://www.geneious.com). The alignment was trimmed to remove primer sequences on the 5′ and 3′ ends, resulting in an alignment of ~178 bp (Figure 1, Step 1). A percent identity (PID) matrix was calculated based on percent similarity of nucleotides in each sequence pair (Figure 1, Step 2), and a PID histogram was generated (Maes et al., 2009; Figure 1, Step 3a).

A two‐step approach was then used to define OTUs using the PID matrix (Figure 1, Step 3a and 3b). First, the number of OTUs was estimated using the two visible PID cutoffs, the values of which are shown as breaks (or troughs) in the PID histogram (Maes et al., 2009; Figure 1, Step 3a). Because multiple troughs exist in the histogram, and to remove the subjectivity involved in determining which trough to use as the cutoff, a second step was introduced to objectively support the use of one cutoff, as opposed to another. In this step, the number of OTUs was determined using affinity propagation via the R package apcluster (Bodenhofer et al., 2011; Figure 1, Step 3b). Affinity propagation is a clustering algorithm that examines all cells in a similarity matrix (e.g., in a PID matrix) to identify exemplars (Frey & Dueck, 2007). In the case of viral sequences, exemplars are single sequences that are the best representatives (akin to type sequences) of an entire cluster, and one exemplar exists per cluster. The remaining sequences are then clustered around the exemplars (see Appendix and Frey & Dueck, 2007).

When inputting the PID matrix, the number of clusters identified through affinity propagation can be interpreted as an optimal number of viral OTUs. Affinity propagation was used in place of other clustering algorithms (e.g., *k*‐means) because the number of clusters (i.e., OTUs) was not a required, a priori input parameter. Although affinity propagation has been used to define clusters of a single viral species (Fischer et al., 2017), this is the first instance in which it has been used to delineate viral units for the purpose of community‐level analyses. To conduct affinity propagation analyses (Bodenhofer et al., 2011), the apcluster() command was run with a single line of code, inputting the PID matrix and using default values of input parameters (input data = NA, input preference = NA, *q* = 0.5, convits = 100, maxits = 1000, lam = 0.9). The results of PID histogram cutoffs and affinity propagation were then used in tandem to classify OTUs (Figure 1, Step 4, see Section 3).

To further explore the utility of PID histograms and affinity propagation as a two‐step means to define appropriate viral OTUs, we gathered from GenBank full‐length DNA polymerase sequences (~ 3000 bp each, representing about 1% of the full herpesvirus genome) for each of 16 established herpesvirus species, as ratified by the ICTV (King et al., 2012; Table A1). Additionally, to explore the method's sensitivity to sequence length, we trimmed the full‐length polymerase sequences of ratified viral species to the same 178 bp region represented by the samples collected in Puerto Rico. We then performed the two‐step method for defining OTUs on these two sets (i.e., full‐length and trimmed) of established species. Further details are included in the Appendix. For ease of exposition, “species” will be used hereafter to reference host taxa and formally described viral taxa, whereas “OTU” will be used to reference an empirical viral taxonomic unit detected in Puerto Rican bats.

To compare OTUs with established herpesvirus species, a maximum likelihood (ML) cladogram was generated. Exemplar sequences, as defined using affinity propagation, were chosen to represent each OTU. Sequences were aligned using ClustalW and executed in Geneious version 11.0.5. ML trees were constructed in Mega7 (Kumar et al., 2018). For the beta subfamily, the best model was Kimura two‐parameter + G + I, and for the gamma subfamily the best model was Hasegawa‐Kishino‐Yano + G + I (Hasegawa et al., 1985; Kimura, 1980). Node support was assessed using 500 bootstraps.

### 2.4 Viral discovery curves

To determine how well the collected samples represent the true, imperfectly sampled metacommunity of herpesviruses infecting bats at Mata de Plátano Nature Reserve, viral discovery curves were created using the iNEXT package in R (Hsieh et al., 2016). By defining the metacommunity as all of the herpesviruses infecting all of the host species at Mata de Plátano Nature Reserve, an assumption is made that herpesviruses are shared between caves and among host species (i.e., no host specificity). Because this may not be a valid assumption, separate curves were generated for each bat species, as well as for each cave. Additionally, Chao2 estimates of richness (Chao, 1987) were calculated for the viral metacommunities of all bats at Mata de Plátano Nature Reserve, as well as for each cave and for each bat species separately.

### 2.5 Rank frequency distributions

Distributions were plotted based on frequency of occurrence (i.e., prevalence; rank frequency distributions, or RFDs) of each OTU in the viral metacommunity at Mata de Plátano, as well as separately for each bat species or cave, and across host individuals. RFDs were used in place of species abundance distributions (SADs) or rank abundance distributions (RADs), as methods did not allow for quantification of viral abundance. Between all pairs of host species and caves, chi‐squared goodness‐of‐fit tests compared the empirical RFDs based on simulated distributions involving 10,000 randomizations (Platefield, 1981) using the chisq.test() command in R. To account for multiple comparisons among RFDs of host species, experiment‐wise error rate was calculated as.

$$1–{\left(1–\alpha \right)}^{1/k}$$

keeping *α* = 0.05 (Sokal & Rohlf, 1995). Pairwise comparisons were made between six host species for *k* = (6*5)/2 = 15.

### 2.6 Host specificity

For each OTU that infected more than one host species, a host specificity index (*S*_TD_*) was calculated (Poulin & Mouillot, 2005) as

$$S_{\mathrm{TD}}^{*}=\frac{\sum \sum_{i<j}\omega_{\mathrm{ij}}\left(p_{i}p_{j}\right)}{\sum \sum_{i<j}\left(p_{i}p_{j}\right)},$$

where *i* and *j* represent index values of host species, *p* represents OTU prevalence within host populations, and *ω*_*ij*_ is the phylogenetic distinctiveness of hosts. To measure phylogenetic distinctness, hosts of the same genus are assigned a value of one, hosts of the same family but different genera are assigned a value of two, and hosts of different families have a value of three (Poulin & Mouillot, 2005). *S*_TD_* thus weighs host preference by the relatedness of hosts (incorporating evolution) and the prevalence of the pathogen in each of its hosts (incorporating ecology). The extent of viral sharing among hosts species was further examined by determining whether particular OTUs had a primary host species. A primary host species was defined as a single host species for which an OTU had significantly higher prevalence than in all other host species combined. To do this, the prevalence of each OTU in its primary host and its combined prevalence in all other hosts were compared using Fisher's exact test (Wassertheil‐Smoller & Smoller, 2015).

To put host specificity of herpesvirus into an ecological context, we next examined host use from an occupancy‐abundance perspective. The number of infected host species was plotted against average prevalence across all host species, and across infected host species, for each viral OTU. In this regard, we used OTU frequency of occurrence (i.e., prevalence, or percent of individuals infected in a host population) as a proxy for abundance (e.g., number of individual virions infecting a host population). We then calculated Pearson's product–moment correlation for each relationship.

All analyses were performed using R version 3.4.3, and code, sequences, and metadata used can be found at github.com/ARSjodin/EcologicalAnalysesHerpesviruses. Sequences and metadata can be additionally found in Dryad (https://doi.org/10.5061/dryad.z8w9ghxcs), and sequences are included in Data S1.

## 3 RESULTS

### 3.1 Viral screening

Oral swabs were collected from 1086 bats representing eight species (*A. jamaicensis* and *Ept. fuscus* in Larvas Cave, and *P. quadridens*, *P. portoricensis*, *Mor. blainvillei*, *Mon. redmani*, *Ero. bombifrons*, and *B. cavernarum* in Culebrones Cave; Table 1). In total, 330 of the 1086 host individuals were positive for herpesvirus, resulting in a community‐level prevalence of 30.4% (Table 1). Prevalence was similar in Culebrones (30.0%) and in Larvas (35.3%) Caves. Herpesvirus prevalence at the host population‐level ranged from 12.9% in *P. portoricensis* to 42.1% in *A. jamaicensis*. At least one individual from each host species tested positive, with the exception of *Ept. fuscus* (0 of 11).

**TABLE 1:** Summary of capture numbers and virus screening results.

Host species	*N*	*N* positive	Prevalence	*S* (% completeness)	*N* common, *N* rare	Chao2 (95% lower, upper)
*Pteronotus quadridens*	288	56	19.44	15 (55)	5, 10	27.207 (17.194, 82.937)
*P. portoricensis*	31	4	12.90	3 (93)	2, 1	3.242 (3.013, 7.622)
*Mormoops blainvillei*	299	120	40.13	9 (67)	3, 6	13.485 (9.493, 49.788)
*Monophyllus redmani*	262	90	34.35	12 (74)	2, 10	16.151 (7.974, 70.975)
*Erophylla bombifrons*	74	12	16.22	7 (47)	3, 4	14.892 (6.361, 30.295)
*Brachyphylla cavernarum*	64	24	37.5	7 (93)	3, 4	7.492 (7.029, 15.332)
Culebrones Cave Total	1018	306	30.06	39 (82)	9, 30	47.325 (41.018, 73.345)
*Artibeus jamaicensis*	57	24	42.11	7 (61)	2, 5	11.421 (7.486, 47.238)
*Eptesicus fuscus*	11	0	NA	NA	NA	NA
Larvas Cave Total	68	24	35.29	7 (61)	2, 5	11.434 (7.487, 47.348)
Grand Total	1086	330	30.39	43 (78)	11, 32	55.089 (46.029, 91.24)

*Note*: Numbers of host individuals sampled (*N*), number testing positive for herpesvirus (*N* positive), and prevalence of herpesviruses are calculated for each host species and for each cave. Number of herpesvirus OTUs detected empirically (*S*) and estimate of herpesvirus OTU richness (Chao2), are compared to estimate the percentage of herpesvirus OTUs that was detected in this study (% completeness). No viruses were detected from *Eptesicus fuscus*, so NA values are produced.

### 3.2 OTU classification

**FIGURE 2:**
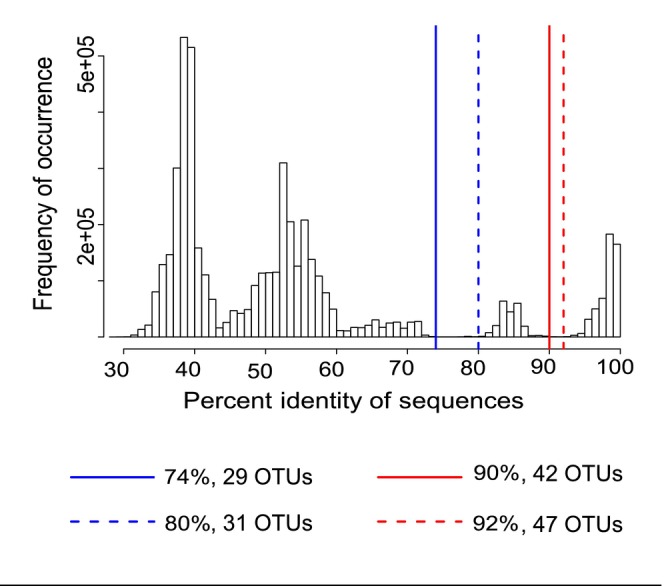
Percent Identity (PID) histogram of herpesviruses calculated using an alignment of all 3131 clones. The x‐axis represents the percent identity of clone sequences compared pairwise. The y‐axis represents the frequency of occurrence. Colored, vertical lines represent cutoff points corresponding to two distinct troughs, with blue lines representing a less specific, cutoff (analogous to genus or subfamily) and the red lines representing a more specific cutoff (analogous to species, subspecies, or strain). Solid lines represent the low end of the cutoff ranges, and the dashed lines represent the upper end of the cutoff ranges. Four distinct PID cutoffs are demonstrated (74%, 80%, 90%, and 92%), resulting in four different numbers of OTUs (29, 31, 42, and 47, respectively).

PCR products from each of the 330 positive host individuals were cloned and sequenced. In total, 3131 sequences were generated from the 330 positive host individuals. To represent these sequences as OTUs for ecological analysis, a PID matrix was generated from a total clone alignment of all 3131 sequences. This resulted in two clearly differentiated potential cutoff ranges, as demonstrated by troughs in the histogram (Figure 2).

Two different approaches were used in combination to define OTUs from the PID matrix. In the first step, the number of OTUs were defined based on cutoffs that reflect breaks in the histogram (Maes et al., 2009). Four possible breaks were identified (blue and red vertical lines in Figure 2), indicating that several PID cutoff values could be used to delineate OTUs. One possible PID cutoff value for these data was 90% (solid red vertical line in Figure 2). This would mean that two sequences with less than 90% genetic similarity would be considered to be different OTUs. Conversely, any two sequences that share ≥90% sequence identity would be considered the same OTU. Other potential cutoff values were 74% (solid blue vertical line in Figure 2), 80% (dotted blue vertical line in Figure 2) and 92% (dotted red vertical line in Figure 2). The number of OTUs resulting from the 74%, 80%, 90%, and 92% cutoffs were 29, 31, 42, and 47 OTUs, respectively.

In contrast, affinity propagation resulted in 45 OTUs. This is within the range of OTUs defined using PID cutoffs at the higher PID values (i.e., 42–47 OTUs at 90%–92% similarity), indicating that the higher PID cutoffs are better supported from a quantitative perspective. A cutoff of 90% is also consistent with the genetic distance between established herpesvirus species within the betaherpesvirus and gammaherpesvirus subfamilies. When the GenBank sequences of ratified herpesvirus species were examined, no two betaherpesvirus or gammaherpesvirus species were greater than 90% similar (but see discussion for a single exception). Together, these results suggest that a cutoff of 90% is a quantitatively and biologically supported PID for classifying OTUs. Using the 90% cutoff, 43 OTUs were detected in empirical samples.

To validate the utility of the PID histogram‐affinity propagation method, we evaluated how it would perform on 107 sequences representing 16 herpesvirus species ratified by the ICTV (mean = 6.69 sequences per species). The results suggest that affinity propagation is not limited by sequence length while using a conserved genome region, such as the polymerase, but is sensitive to the number of sequences available for each cluster. See Appendix for further details.

OTU richness per host population ranged from three to 15. For comparison, humans are the only host species for which comprehensive data are available on herpesvirus richness, and only eight known herpesvirus species infect people, despite the cosmopolitan distribution of humans. OTU richness per host individual ranged from one to four. Seventy‐eight individuals were coinfected with more than one OTU: 67 individuals had two OTUs, 10 individuals had three OTUs, and one individual had four OTUs.

**FIGURE A1:**
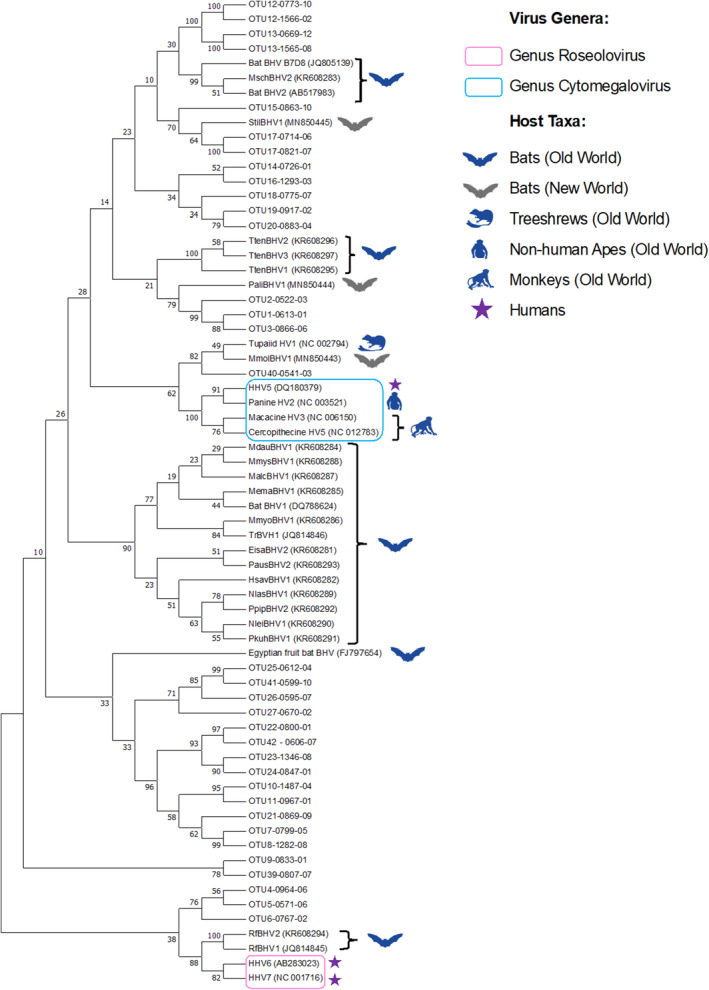
Cladogram for the Betaherpesvirinae (BHV), generated using the Kimura two‐parameter + G + I model. Hosts of previously discovered viruses are coded blue for Old World and gray for New World distribution. Purple stars indicate human viruses (HHV), and only those sequences that have been officially classified into genera ratified by the ICTV are highlighted in color. GenBank accession numbers for these sequences are listed in parentheses. All sequences beginning in “OTU” were sampled from Puerto Rican bats in this study, with the following four‐ and two‐number codes representing sample ID and clone number (01–12), respectively. Sequences chosen to represent OTUs were all exemplars of their respective clusters. The cladogram was generated using a short, 172 base pair alignment, so any inferences should be made with caution. Sequence references for bat hosts: Wibbelt et al. (2007), Jánoska et al. (2011), Pozo et al. (2016), and James et al. (2020).

**FIGURE A2:**
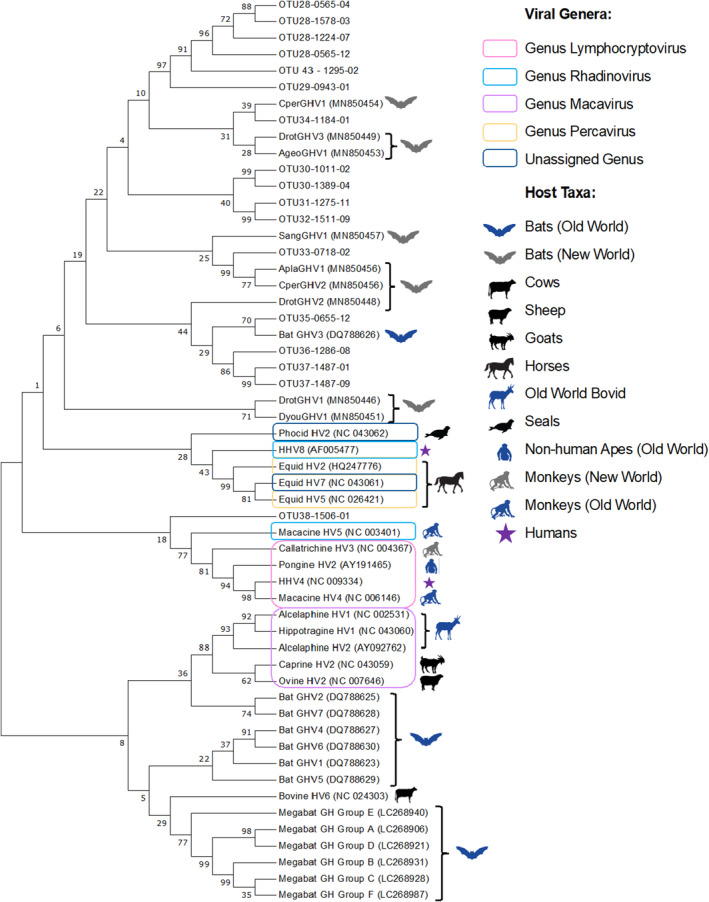
Cladogram for the Gammaherpesvirinae (GHV), generated using the GKY + G + I model. Hosts of previously discovered viruses are coded blue for Old World, gray for New World, and black for global (i.e. domesticated) distribution. Purple stars indicate human viruses (HHV), and only those sequences that have been officially classified into genera ratified by the ICTV are highlighted in color. GenBank accession numbers for these sequences are listed in parentheses. All sequences beginning in “OTU” were sampled from Puerto Rican bats in this study, with the following four‐ and two‐number codes representing sample ID and clone number (01–12), respectively. Sequences chosen to represent OTUs were all exemplars of their respective clusters. The cladogram was generated using a short, 169 base pair alignment, so any inferences should be made with caution. Sequence references for bat hosts: Wibbelt et al. (2007), Shabman et al. (2016), Wada et al. (2018), and James et al. (2020).

Cladograms were generated using maximum likelihood for the Beta and Gamma subfamilies and had overall low bootstrap support (Figures A1 and A2, respectively). For both subfamilies, OTUs were generally most closely related to other viruses with bat hosts, although caution should be exercised when interpreting relationships due to the short, ~175 bp sequence lengths and the low bootstrap support. In this context, the cladograms should be used to illustrate degree of genetic separation between lineages, rather than the accurate reconstruction of shared ancestors.

### 3.3 Viral discovery curves

**FIGURE 3:**
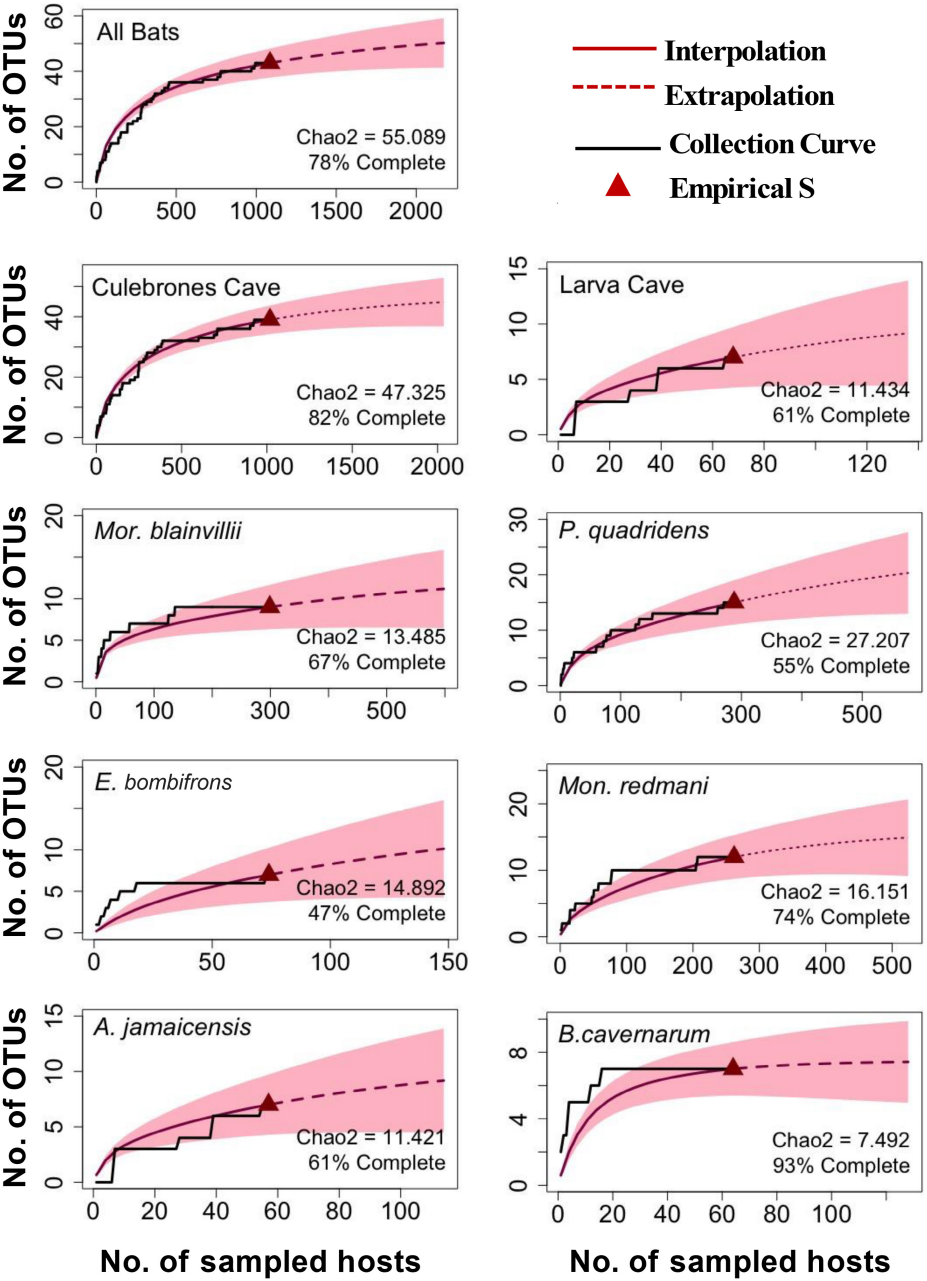
Viral discovery curves comparing number of detected herpesviruses (OTU richness) with number of sampled host bats for all bats at Mata de Plátano Nature Reserve (top panel), for each cave (middle two panels), and for each host species (bottom six panels). The black lines represent the empirical pattern of OTU accumulation. The dashed red lines represent extrapolation of undiscovered herpesviruses in the metacommunities, with red shading demonstrating error associated with the estimates. Empirical viral richness per metacommunity (*S*) is depicted by red triangles. Chao2 numbers estimate true viral richness in each community and are used to calculate the percent of total estimated OTUs that were detected in samples (percent completeness). “Number” is abbreviated as “No.”.

The Chao2 estimate of herpesvirus richness in the bats of both caves was 55.1 OTUs (Table 1). As such, the detected OTUs (*N* = 43) represent about 78% of those in the entire herpesvirus metacommunity (Figure 3). Viral discovery curves were more saturated in Culebrones (82%) than in Larvas (61%) Cave, and host species‐level saturation (Table 1; Figure 3) ranged from 47% (*Ero. bombifrons*) to 93% (*B. cavernarum*). A viral discovery curve was not generated for the herpesvirus metacommunity infecting *P. portoricensis*, as only three OTUs were detected.

### 3.4 Rank frequency distributions

**FIGURE 4:**
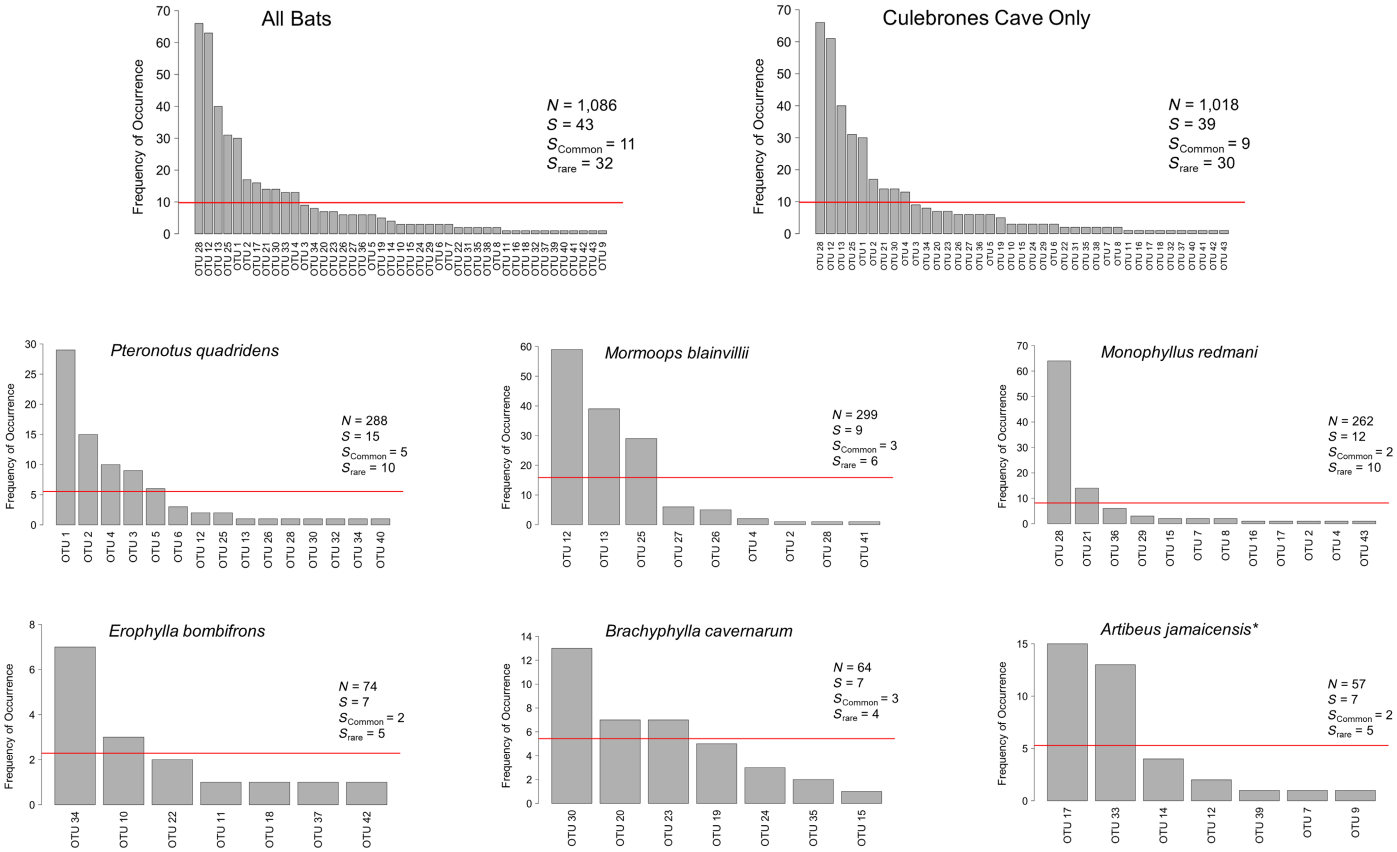
Rank frequency distributions for all bats, for Culebrones cave, and for each individual host species. OTUs are listed on the x‐axis ranked from most frequent (i.e., common) to least frequent (i.e., rare). The y‐axis represents the frequency of occurrence (i.e., prevalence), or the number of hosts in which the OTU was detected. OTUs with frequencies of occurrence at or above the red lines (1/*S*_Total_) represent common OTUs, whereas those below the red lines represent rare OTUs. Total hosts sampled (*N*), number of OTUs detected (*S*), and number of common (*S*_common_) and rare (*S*_rare_) are shown for each viral community. *A. jamaicensis* represents the only species not found in Culebrones Cave, as demonstrated by the *.

The herpesvirus metacommunity infecting bats of Mata de Plátano Nature Reserve exhibits a long‐tailed distribution with few common and many rare OTUs (Figure 4). When examined at the scales of cave and host population, viral metacommunities showed similarly right‐skewed patterns (Figure 4). At the scale of host population, after accounting for experiment‐wise error rate, RFDs were significantly different only between *Mor. blainvillei* and *Mon. redmani* (*p* = .002; Table 2). Pairwise comparisons of RFDs between all other species, and between caves, were not significant (Table 2). Because only three OTUs were detected in *P. portoricensis*, the RFD for this species was not compared to those of other species. Additionally, the RFD for Larva Cave is identical to that of *A. jamaicensis*, as this was the only host species roosting in Larva Cave that tested positive for any viruses.

**TABLE 2:** Pairwise comparisons of rank frequency distributions (RFDs) between caves and host populations based on chi‐squared (*X* ^2^) analyses.

		*X* ^2^	*p*‐Value
Between Caves			
Culebrones	Larvas	30.39	.713
Between populations			
*Pteronotus quadridens*	*Mormoops blainvillei*	23.43	.019
*Pteronotus quadridens*	*Monophyllus redmani*	21.65	.043
*Pteronotus quadridens*	*Erophylla bombifrons*	3.21	.998
*Pteronotus quadridens*	*Brachyphylla cavernarum*	5.19	.999
*Pteronotus quadridens*	*Artibeus jamaicensis*	9.25	.901
*Mormoops blainvillei*	*Monophyllus redmani*	25.21	**.001**
*Mormoops blainvillei*	*Erophylla bombifrons*	6.83	.453
*Mormoops blainvillei*	*Brachyphylla cavernarum*	10.21	.247
*Mormoops blainvillei*	*Artibeus jamaicensis*	4.10	.848
*Monophyllus redmani*	*Erophylla bombifrons*	6.00	.755
*Monophyllus redmani*	*Brachyphylla cavernarum*	19.56	.030
*Monophyllus redmani*	*Artibeus jamaicensis*	11.90	.361
*Erophylla bombifrons*	*Brachyphylla cavernarum*	1.46	.988
*Erophylla bombifrons*	*Artibeus jamaicensis*	2.18	.963
*Brachyphylla cavernarum*	*Artibeus jamaicensis*	5.37	.537

*Note*: Keeping the experiment‐wise error rate at .05, individual *p*‐values for each pairwise comparison of host populations are considered significant, and bolded, at a value of less than or equal to .003.

### 3.5 Host specificity

Thirteen OTUs were detected in more than one host species (Table 3), and the remaining 30 OTUs were each found in a single host species. *S*_TD_* for each of the shared OTUs ranged from one (infecting two hosts from the same genus) to three (infecting three hosts of two families). Nine OTUs had significantly higher prevalence in one host species than in all other host species combined.

**TABLE 3:** Host specificity index (*S*_TD_*) for each of the 13 herpesvirus OTUs that infect more than one host species.

OTU	Species infected	Viral sub‐family	*S*_TD_*	*P. qua* (*N* = 288)	*P. por* (*N* = 31)	*Mor. bla* (*N* = 299)	*Mon. red* (*N* = 262)	*E. bom* (*N* = 74)	*B. cav* (*N* = 64)	*A. jam* (*N* = 57)	1°/N	2°/N	*p*‐Value
1	2	Beta	1.00	**0.101**	0.032	0.000	0.000	0.000	0.000	0.000	29/288	1/31	.210
2	3	Beta	2.55	**0.052**	0.000	0.003	0.004	0.000	0.000	0.000	15/288	1/561	<.001**
4	3	Beta	2.41	**0.035**	0.000	0.007	0.004	0.000	0.000	0.000	10/288	3/561	.002*
7	2	Beta	2.00	0.000	0.000	0.000	0.008	0.000	0.000	**0.018**	2/57	1/262	.920
12	3	Beta	2.84	0.007	0.000	**0.197**	0.000	0.000	0.000	0.035	59/299	4/345	<.001**
13	2	Beta	2.00	0.004	0.000	**0.130**	0.000	0.000	0.000	0.000	39/299	1/288	<.001**
15	2	Beta	2.00	0.000	0.000	0.000	0.008	0.000	**0.016**	0.000	2/64	1/262	.900
17	2	Beta	2.00	0.000	0.000	0.000	0.004	0.000	0.000	**0.263**	15/57	1/262	<.001**
25	2	Beta	2.00	0.007	0.000	**0.097**	0.000	0.000	0.000	0.000	29/299	2/288	<.001**
26	2	Beta	2.00	0.004	0.000	**0.017**	0.000	0.000	0.000	0.000	5/299	1/288	.120
28	3	Gamma	2.99	0.004	0.000	0.003	**0.248**	0.000	0.000	0.000	65/262	2/587	<.001**
30	2	Gamma	3.00	0.004	0.000	0.000	0.000	0.000	**0.188**	0.000	12/64	1/288	<.001**
34	2	Gamma	3.00	0.004	0.000	0.000	0.000	**0.095**	0.000	0.000	7/74	1/288	<.001**

*Note*: OTU prevalence is calculated for each host species, with number of sampled individuals in parentheses (*N*). Prevalence in the primary host species is bolded for each OTU. Number of primary hosts sampled is indicated by 1°, and number of secondary hosts sampled is indicated by 2°. Comparison of OTU prevalence between primary host species and all other host species for which it is found used Fisher's exact tests. Significance at an alpha of .01 are marked with a single asterisk, whereas those significant at an alpha of .001 are marked with two asterisks.

**FIGURE 5:**
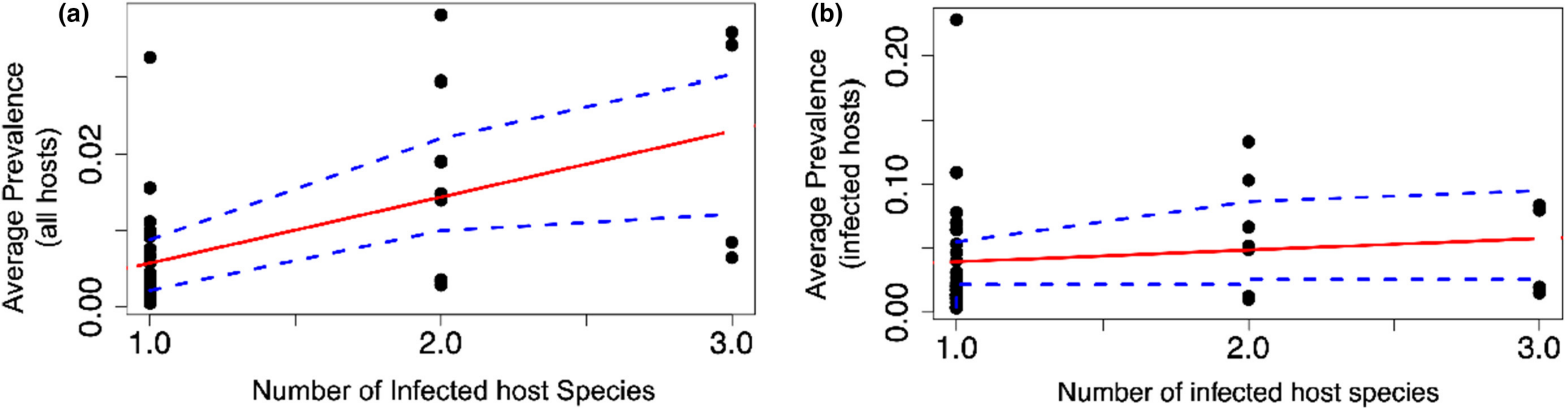
Abundance‐occupancy relationships of herpesvirus OTUs. Abundance is averaged over (a) all bat species (*r* = .544, *p* < .001) and (b) only those species infected with the OTU of interest (*r* = .135, *p* = .389). Linear trends and 95% confidence intervals for each relationship are shown in red solid and blue dashed lines, respectively.

When infection prevalence across all species, regardless of infection status, was compared to the number of hosts infected for each OTU, a significant, positive correlation emerged (*r* = .544, *p* < .001; Figure 5a). However, when the relationship was examined across only those species testing positive for each OTU, the correlation was not significant (*r* = .135, *p* = .389; Figure 5b).

## 4 DISCUSSION

An ecological community can be defined as two or more taxa sharing the same space at the same time, with the potential for interaction (Begon et al., 1996; Mittelbach & McGill, 2019). Therefore, to study ecological communities, distinct, replicable operational units, or taxa, must be classified. Although community ecology can be studied using various taxonomic levels (e.g., genus; Bergner et al., 2020), historical principles were developed using species as the unit of interest, as species were thought to represent reproductively isolated evolutionary units. Officially delineating viral species, however, is time consuming and systematically conducted by committee, mostly requiring full genome sequences and oftentimes requiring additional functional or biological data (Adams et al., 2013; Van Regenmortel et al., 2013). Consequently, OTUs are commonly used as species surrogates. Although multiple methods exist for delineating OTUs (e.g., histograms using percent genetic identity: Maes et al., 2009; monophyletic groups: Anthony et al., 2015; or genetic divergence: Lauber & Gorbalenya, 2012; Muhire et al., 2014), subjectivity is generally required to choose the best cutoff in terms of genetic similarity (i.e., the histogram method) or evolutionary distance (i.e., the monophyletic groups method). Although subjectivity is not required in the case of using genetic divergence (Lauber & Gorbalenya, 2012, Muhire et al., 2014), the methods were developed specifically using full genomes, or the longest possible sequences, and they require use of multiple programs or coding languages.

### 4.1 Taxonomic categorization

Affinity propagation is a machine‐learning algorithm developed to cluster a broad range of data (e.g., images of faces, genes in a microarray, heavily traveled cities) and to identify representative examples of each underlying cluster (Frey & Dueck, 2007). The method has been used to cluster strains of rabies viruses (Fischer et al., 2017) but has never been used to identify viral OTUs for analyses in the context of community ecology. Here, we explore the utility of affinity propagation within the context of a molecularly superficial ecological survey of herpesvirus diversity. We integrate affinity propagation with percent identity histograms to classify short viral sequences into robust OTUs that we then use to explore patterns of co‐infection and viral sharing among co‐roosting host species. Although this method does not contain the depth and quantity of information that would be available if each OTU went through the ICTV process to define official species, it is a reasonable, quickly implemented method that can be used to reliably and objectively demonstrate ecological patterns until completing further taxonomic work. Additionally, this two‐step methodology could be easily applied to any measure of similarity for which a PID matrix can be calculated, including amino acid sequence as well as phylogenetic or functional similarity.

Using a combined approach, we delineated 43 herpesvirus OTUs in 330 bats of seven species. The extra step of affinity propagation used the same data input as the first step of histogram generation, required only a single line of code, and took less than three minutes to implement. Additionally, we showed that OTU delineation was consistent with current taxonomic classifications of known herpesvirus species, suggesting that this combined approach has considerable biological and practical merit. Importantly, we do not suggest this method as replacement for the more in‐depth, holistic viral classification conducted by the ICTV. Instead, we show that it can serve as a biologically relevant and quantitatively‐supported method to cluster novel viral sequences that have not yet been fully investigated by the ICTV. We recognize that, with time, and through the iterative process of science, the exact species limits of the viruses evaluated here may differ from OTU delineations.

Importantly, we do not advocate for affinity propagation as a “stand‐alone” method for clustering viral sequences into OTUs. Rather, we suggest using it to provide an objective, quantitative support for delineating clusters based on a chosen comparative metric, such as genetic or phylogenetic relatedness. Here we combined affinity propagation with PID histograms and note that our ability to define cutoffs would be made more difficult if discrete troughs were not observed in the PID histograms. Clear troughs have also been identified in other viral taxa (e.g., Hantaviruses; Maes et al., 2009), showing that there would be utility to our combined approach beyond this study. Affinity propagation could also be easily applied alongside monophyletic grouping when robust phylogenies are available.

### 4.2 Integrating viral ecology with community ecology

Although this methodology, and thus this work, does not identify novel viruses, we show progress in integrating viral ecology with community ecology, as both fields involve focusing on emergent properties of a profile of taxonomic units, instead of concerning single entities, the identity and function of which may be unknown (Jurburg et al., 2022; McGill et al., 2007). Microbial ecologists facing a similar lack of specific bacterial identities have advanced their field by emphasizing compositional data, relative analyses such as ratios, and global associations between community structure and phenotypes of interest (Gloor et al., 2016; Jurburg et al., 2022; Pawlowsky‐Glahn et al., 2015; Rivera‐Pinto et al., 2018). Therefore, we are confident that descriptions of herpesvirus communities that lack properly identified species can still provide foundational data to inform the nascent field of viral community ecology.

Species accumulation curves for herpesviruses show that the sequence diversity detected in this study is incomplete. At the largest scale (i.e., the entire bat community), sampling completeness reached 80%. This is in contrast to accumulation curves for viral metacommunities infecting individual host populations, which were as low as 55% (*P. quadridens*). This suggests that undiscovered OTUs at the scale of host population have already been discovered at the scale of host community (i.e., have already been discovered in another host species). This supports the hypothesis that herpesviruses are shared among host species, and that certain OTUs that are common in a primary host are rare in all other host species combined.

As is well supported in many free‐living organisms (Magurran & Henderson, 2011; McGill et al., 2007), microbes (Goldford et al., 2018; Hill et al., 2003), and macroparasites (Poulin et al., 2008), metacommunities of herpesviruses comprised few common and many rare OTUs, regardless of focal scale (i.e., host individuals, host species, or host community). For abundance distributions, a rich ecological literature exists comparing empirical distributions to various models as a way to identify mechanisms that underlie such “laws” (McGill et al., 2007). Dozens of comparison models exist, ranging from purely statistical, to those guided more strongly by ecological hypotheses about assembly mechanisms (McGill et al., 2007). The theory guiding model choice for viral communities is not yet established. Additionally, RADs or SADs could not be generated here, as viral abundance was not measured. Conclusions can only be drawn regarding *frequency* distributions (RFDs), and as such, results should be interpreted with caution. Nonetheless, we show that viruses may follow well‐established ecological “laws” that are characteristic of free‐living and macroparasitic organisms (McGill et al., 2007; Poulin et al., 2008), supporting the use of ecological perspectives to better understand viral dynamics.

### 4.3 Structuring mechanisms

One mechanism that could be at play in shaping the long‐tailed RFD for herpesviruses is temporal turnover of OTUs resulting from viral latency. Although the lack of temporal turnover of OTUs is a negligible factor in herpesvirus detection efforts, it is likely still at play at a timescale coincident with viral community assembly. Studies of free‐living organisms may attribute temporal turnover to immigration or local extinction (MacArthur & Wilson, 1967), but in this system, temporal turnover also arises from changes in viral activity. Sampling methods were non‐invasive‐detected viruses represented only those being actively shed by bats at the time of sampling. They did not account for infections in their latent phase. Latency and lifelong infection could also contribute to maintenance of rare OTUs, as extreme longevity may allow currently rare viruses to be common at other times (Wisnoski et al., 2019), as has been documented in some plants (Magurran & Henderson, 2011). Additionally, dormancy, a similar process as latency in its reversible state of reduced metabolic activity, has shown strong effects on microbial diversity and distributions (Lennon & Jones, 2011; Locey, 2010; Locey et al., 2020), providing support for latency as a mechanism that contributes to the form of RFDs in herpesvirus communities.

Viral infection of dead‐end hosts could be another mechanism that shapes long‐tailed RFDs in herpesviruses. These are instances in which a pathogen can spill over and infect a new host species but cannot further transmit from this host. Consequently, the pathogen does not establish positive population growth or emergence in the new host species. This could account for single occurrence OTUs in a host population and is analogous to a free‐living organism captured in a sub‐optimal habitat through which it passes without establishing. Comparisons of OTU prevalence between primary and secondary hosts shows that most herpesvirus OTUs have a core host, supporting the possibility that dead‐end hosts may exist in these metacommunities.

Finally, left‐skewed RFDs could be attributed to the chosen definition of rarity. Because the RFDs are based on frequency of OTU occurrences, viruses that are considered rare here could exist in high abundances within hosts. This phenomenon could be explored by developing quantitative PCR assays for each viral OTU, allowing a measure of viral intensity in each sample. Recognizing the differences between the use of frequency of occurrence versus viral abundance will be of continued importance in further studies using rank distributions to understand viral community assembly.

The significant difference in RFDs between *Mor. blainvillei* and *Mon. redmani* suggest that herpesvirus communities infecting different host species may be structured via different assembly mechanisms. For example, the richer viral community of *Mon. redmani* (*S* = 12, Chao2 estimate = 16.151; Table 1) may be more strongly shaped by positive interactions among OTUs, with viruses facilitating co‐infection (Poulin, 2007). Conversely, the less rich community in *Mor. blainvillei* (*S* = 9, Chao2 estimate = 13.485; Table 1), may be dominated by competitive interactions among OTUs (Poulin, 2007) or priority effects through activation of the host immune system. Although significant differences did not exist between most species, this could reflect under‐sampling of populations. Future studies should investigate viral interactions and host heterogeneity within the context of RFDs or SADs to examine these hypotheses.

### 4.4 Host specificity

Our primary hypothesis was that herpesviruses would show significant associations with particular bat species. We further hypothesized that, despite associations with a primary host species, certain viruses would also be identified in co‐roosting species of bats due to the proximity of host individuals and the opportunity for viral spillover within caves. We found that nine of 13 multi‐host OTUs in our study had significantly higher prevalence in a single host species than in all other hosts in which it occurred. This shows that in this system, herpesviruses are indeed associated with a single, primary host species and that cross‐species transmission occurs regularly. Such regular viral sharing has implications for long‐term viability of the viruses, as it facilitates the establishment of new host‐virus associations after changes or disturbances to host ecology (Brooks & Ferrao, 2005; Brooks & Hoberg, 2007; Hoberg & Brooks, 2008). This is particularly important in places such as Puerto Rico, where hurricane disturbance is a defining feature of the ecosystem. In Culebrones cave, specifically, hurricane Georges caused shifts in the composition of bat species (Rodríguez‐Durán, 2009). Prior to Georges, *Ero. bombifrons* was the most abundant species in Culebrones, whereas after Georges, at the time of sampling, the community was dominated by *P. quadridens*, *Mor. blainvillei*, and *Mon. redmani*.

Various factors, including the ecology, behavior, and phylogenetic relatedness of hosts, can suggest why viruses may be shared among particular species. For example, nonrandom associations are common among cave dwelling bats in Puerto Rico, most likely relating to eco‐physiological characteristics of the bats, patterns of activity, and morphology of caves (Rodríguez‐Durán, 1995, 1998). In hot caves, like Culebrones, the strongest roosting associations occur among species belonging to two groups, those roosting in the Caldarium and those roosting in the Tepidarium (Rodríguez‐Durán, 2020). *Mon. redmani*, *P. quadridens*, and *Mor. blainvillei* all roost in the Caldarium, the hottest portion of the cave, whereas the cooler Tepidarium is home to *Ero. bombifrons*, *P. portoricensis*, and potentially *B. cavernarum* (Rodríguez‐Durán, 2020). Species within each of the two groups typically maintain spatial separation while sleeping, but all species from both groups use additional chambers (e.g., a waiting chamber prior to cave egress) while awake, where they may occasionally come into contact or interact while moving about (Silva‐Taboada, 1983). This spatial separation is sometimes violated, as individuals of one species can be found embedded in a group of bats of a different species. Such regular physical proximity provides an increase in opportunities for transmission and likely contributes to the interspecific sharing of viruses such as OTUs 2, 4, and 28, which were each detected in all three Caldarium species (Table 3). Additionally, the large numbers of bats that roost together in Culebrones forces many individuals to travel long distances to access foraging locations during the night. Bats have multiple feeding bouts throughout the night and often use temporary night roosts between such bouts, rather than travel back to their day roosts. These night roosts may include entrances of other caves, and indeed *Ero. bombifrons* has been observed using Larvas cave as a temporary night roost. This sort of temporary roosting behavior may facilitate interspecific contact and play a role in viral sharing between caves, as is seen with OTUs 7, 12, and 17 (Table 3). Phylogenetic relatedness of hosts likely also contributes to viral sharing, supporting what may be considered an emerging law in virus ecology (Albery et al., 2021). Hosts of OTUs 1, 7, 15, and 17, for example, belong to the same genus or family. More than likely, however, herpesvirus sharing among Puerto Rican bats follows predicted patterns of viral sharing in mammals (Albery et al., 2020) and is a multi‐faceted effect of all of the above factors.

### 4.5 Community structure

The community‐level patterns of herpesviruses infecting Puerto Rican bats are non‐random, as has been reported for viral communities in other host taxa (e.g., Anthony et al., 2015) and for oral microbiota in this same community of bats (Presley et al., 2021). Significantly positive abundance‐occupancy patterns suggest that these viral communities follow similar assembly rules as do communities of macro‐organisms. Positive abundance‐occupancy relationships, or the observation that abundant species tend to be widespread while rare species have limited geographic ranges, is one of the oldest documented patterns in macroecology (Brown, 1984; Darwin, 1859; Gaston, 1999; Hanski, 1982). Although the patterns can manifest via either neutral or deterministic processes, and data can support multiple co‐occurring mechanisms (Gaston et al., 2000; Verberk et al., 2010; Warren & Gaston, 1997), the results have far‐reaching implications for ecological applications such as biodiversity monitoring (e.g., how many sites must be visited, or hosts must be sampled, to comprehensively describe the ecological community) and expanding habitat (or host) use (Gaston, 1999).

### 4.6 Limitations

Many theories and rules of community ecology and evolution, including those developed to interpret occupancy‐abundance patterns, require counting both species (i.e., OTUs) *and* individuals (i.e., abundance; Brown, 1995; Lawton, 1999). However, the number of virions, or viral particles, which can be thought of as virus “individuals”, cannot be counted as can distinct, macro‐organismal entities (Burrell et al., 2017; Heider & Metzner, 2014). As a result, viral ecology often relies on prevalence, as demonstrated here, and relying on prevalence assumes that viral load is equal in each sampled host. This remains a major criticism of the field of micro‐ecology (Shade et al., 2018), as the extent to which theories based on abundance can be applied to systems for which only prevalence is measured is unknown. A comprehensive assessment of the limitations of studying viral ecology without counting individuals, in the context of established ecological theories, would advance both viral ecology, and ecological theory more generally.

Another limitation of our study concerns the short sequences generated by the herpesvirus cPCR. Short sequences can stymie reconstruction of reliable phylogenetic relationships, evolutionary histories (e.g., identify recombination events; Weller & Sawitzke, 2014), or co‐phylogenetic relationships that would more robustly inform host specificity (e.g., Balbuena et al., 2013) or processes driving symbiont diversification (e.g., McKee et al., 2016). However, cPCR remains an important method for characterizing unknown viral diversity in wildlife despite its inability to produce a full genome or coding sequence. Indeed, in some instances it can be more successful at measuring viral diversity than can deep sequencing approaches (i.e., picobirnaviruses in Anthony et al., 2015). Sequencing the full genome of constituent viruses, or at least the complete coding sequence for multiple genes under different evolutionary constraints, will provide invaluable data for future analyses, but the work presented here demonstrates that such data need not limit the investigation of all questions regarding virus ecology. We find this demonstration to be particularly useful, as one of the most comprehensively sampled datasets of global viral diversity was recently made public, the majority of sequences from which were only available from short, cPCR fragments (PREDICT Consortium, 2020, https://data.usaid.gov). This new body of data is ripe for catalyzing a revolution of thought, a fundamental aspect of scientific discovery (Goldenfeld & Woese, 2007), and we are only just scraping the surface of how such sequence data can guide future understanding. Thus, finding ways to utilize cPCR data in ecological studies is important for advancing the field, exploiting currently available datasets, and deepening the understanding of viruses from an ecological perspective.

Despite the limitation of short sequences, our methods for delineating herpesvirus sequences into OTUs are reasonably robust. Although the goal of our study was not to define viral species, affinity propagation clustering correctly differentiated most established herpesvirus species, even when polymerase sequences were trimmed to only ~178 base pairs. However, this was only true if the number of sequences used per species was >2. The only two species that could not be differentiated (EHV‐1 and EHV‐9) share >97% sequence identity in the polymerase gene and 86–95% identity genome‐wide (Fukushi et al., 2012). Thus, although the short ~175 bp fragment is sufficient to distinguish most herpesviruses (at least, known herpesviruses), it is not able to separate viruses that are closely related in the polymerase gene but divergent in other parts of the genome. It is important that our results be interpreted in light of this limitation, as it is possible that some herpesvirus sequences assigned to the same OTU actually represent different viruses based on other genomic regions.

A final limitation of our study is that our methods do not detect herpesviruses that latently infect tissues other than oral epithelia. Latent viruses are, by definition, not actively reproducing, and therefore presumably do not interact with actively replicating viruses, at least not directly. However, indirect interactions of latent viruses via the immune system may have priority effects on community assembly and consequently play an important role. Additional studies are required to clarify the role of latency on community assembly of herpesviruses.

## 5 CONCLUSIONS

This study advances the field of viral ecology in two ways. First, it presents a novel, two‐step approach to clustering viral sequences using data that are imperfect but also most readily available. In so doing, it facilitates community‐level analyses of the factors shaping viral communities beyond its narrow application in this study system. Second, this work applies the two‐step method to investigate the drivers of herpesvirus diversity in cave‐dwelling bat communities, showing that these viruses have strong host‐associations and are likely structured, to some extent, via non‐random processes. We suggest additional hypotheses that, to the extent of which they can be addressed in future work, represent an opportunity to uncover rules of life that transcend taxonomic silos to unite the fields of viral, microbial, and macrobial ecology. Moreover, studies of the ecology of a taxon that is generally rare and episodically abundant may provide insights that are relevant beyond virus‐host systems.

## AUTHOR CONTRIBUTIONS

**Anna R. Sjodin:** Conceptualization (lead); data curation (lead); formal analysis (lead); funding acquisition (equal); investigation (lead); methodology (lead); project administration (lead); resources (equal); visualization (lead); writing – original draft (lead); writing – review and editing (equal). **Michael R. Willig:** Conceptualization (supporting); formal analysis (supporting); funding acquisition (supporting); investigation (supporting); methodology (supporting); resources (equal); supervision (equal); writing – original draft (supporting); writing – review and editing (equal). **Armando Rodríguez‐Durán:** Conceptualization (supporting); investigation (supporting); resources (supporting); supervision (supporting); writing – review and editing (supporting). **Simon J. Anthony:** Conceptualization (equal); formal analysis (supporting); funding acquisition (equal); investigation (equal); methodology (equal); resources (equal); supervision (equal); writing – original draft (supporting); writing – review and editing (equal).

## CONFLICT OF INTEREST STATEMENT

The authors declare no competing interests.

### OPEN RESEARCH BADGES

This article has earned an Open Data badge for making publicly available the digitally‐shareable data necessary to reproduce the reported results. The data is available at https://doi.org/10.5061/dryad.z8w9ghxcs.

## Supporting information


Data S1

## Data Availability

Genetic sequences and complete dataset are available at https://github.com/ARSjodin/EcologicalAnalysesHerpesviruses and on Dryad at: https://doi.org/10.5061/dryad.z8w9ghxcs. Sequences have additionally been included as Data S1 with this publication.

## Methods

### Affinity propagation

Having determined the optimum number of exemplars, the affinity propagation algorithm clusters the remaining data around the exemplars by iteratively exchanging two types of “messages” between data points. In the context of viral sequences, the first type of message, responsibility or *r*(*i*, *k*), accumulates evidence for sequence *k* as an exemplar for sequence *i*, based on the genetic distance between sequences *k* and *i* (Frey & Dueck, 2007). The second type of message, availability or *a*(*i*, *k*), reflects how many sequences besides *i* support *k* as the best choice of the exemplar (Frey & Dueck, 2007). Responsibility can be viewed as the log‐probability that *k* is an exemplar for sequence *i*. Availability can be viewed as the number of sequences in addition to *i* that support *k* as the best choice of the exemplar. Availability is initialized at zero (i.e. no sequences support *k* as the exemplar), and responsibility is updated according to equation (1) in Frey and Dueck (2007).

### Two‐step OTU delineation with ratified herpesvirus species

For each of the 16 ratified herpesvirus species, we acquired two to 15 non‐identical sequences from GenBank (Table A1). Full polymerase sequences were aligned by ClustalW using the R packages Biostrings (Pages et al., 2017) and msa (Bodenhofer et al., 2015) for an alignment of 2957 bp in length (Figure 1, Step 1). For comparison with our empirically sampled sequences, this alignment was then trimmed to the same ~178 base‐pair region amplified by the Van Devanter et al. (1996) assay (Figure 1, Step 1). For both the full polymerase sequence alignment and the alignment of the trimmed ~178 base‐pair region of ratified species, PID matrices were created (Figure 1, Step 2). We then generated PID histograms (Figure 1, Step 3a) and performed affinity propagation (Figure 1, Step 3b) for each alignment. For the trimmed alignment of known herpesvirus species, affinity propagation was repeated 25 times to determine whether the clustering was consistent among runs. To better understand the sensitivity of affinity propagation to the number of sequences (replicates) per species (i.e., sensitivity to data availability), the method was repeated using the ~178 base‐pair alignment, each time removing sequence replicates from the alignment, until only one replicate represented each known species.

## Results

### Two‐step OTU delineation with ratified herpesvirus species

Using the trimmed alignment of ~178 bp, affinity propagation successfully differentiated nearly all sequences into clusters that reflected their assigned species (Table A2). The only exception was that affinity propagation was never able to differentiate sequences representing equid herpesvirus 1 (EHV‐1) and equid herpesvirus 9 (EHV‐9), two alphaherpesviruses that share >97% identity in the polymerase gene. For the remaining 10 runs, the only species that were not correctly differentiated had fewer than three sequences. When we reduced the number of sequence replicates for each established virus species to determine the lowest number of sequences that were required for accurate clustering, we successfully categorized beta‐ and gammaherpesviruses into their correct species with as few as two sequences per species (Table A3), whereas five sequences per species were needed to differentiate alphaherpesviruses (again, with the exception of equid herpesviruses 1 and 9).

**TABLE A1:** Sequences of established herpesvirus species used to explore the utility of affinity propagation as a means to define appropriate viral OTUs.

Subfamily	Species	Number of sequences	GenBank accession numbers
Alphaherpesvirinae	Human Herpesvirus 1	15	AB070847, AB070848, AB231458, AB231459, AB231460, AB691131, AB691132, AB691133, AB691551, AB691552, AB691550, HQ123151, HQ123153, HQ123179, NC_001806
Alphaherpesvirinae	Human Herpesvirus 2	15	AY038366, AY038367, HQ123175, HQ123176, HQ123177, JQ352198, JQ352199, JQ352200, JX905318, KF588403, KF588404, KF588405, KM068900, KM068901, KU886303
Alphaherpesvirinae	Human Herpesvirus 3	15	AB059829, AB059830, AB059831, KU529486, KU529493, KU529507, KU529515, KU529522, KU529537, KU529538, KU529539, KU529551, KU529553, KU529562, NC_001348
Alphaherpesvirinae	Suid Herpesvirus 1	2	KP279684, NC_006151
Alphaherpesvirinae	Felid Herpesvirus 1	2	AJ224971, NC_013590
Alphaherpesvirinae	Equid Herpesvirus 1	11	KC924817, KC924818, KC924821, KF434378, KF434381, KF434382, KF515985, MN688628, MN688629, MN688630, NC_001491
Alphaherpesvirinae	Equid Herpesvirus 4	4	KF434387, KF434385, KF434486, NC_001844
Alphaherpesvirinae	Equid Herpesvirus 9	3	KX101094, KX101096, NC_011644
Alphaherpesvirinae	Gallid Herpesvirus 1	2	L40431, NC_002229
Alphaherpesvirinae	Gallid Herpesvirus 2	2	MH939248, NC_002577
Betaherpesvirinae	Human Herpesvirus 5	12	AB329634, AB536875, AF133618, AF133619, AF133622, AF291828, AY327256, DQ180362, DQ180379, DQ180391, MK867377, NC_006273
Betaherpesvirinae	Human Herpesvirus 6	4	AB283023, AB283024, NC_000898, NC_001664
Betaherpesvirinae	Suid Herpesvirus 2	6	AF268040, AF268041, HQ113116, HQ686080, HQ686081, MG696113
Gammaherpesvirinae	Human Herpesvirus 4	2	NC_007605, NC_009334
Gammaherpesvirinae	Human Herpesvirus 8	2	AF005477, NC_009333
Gammaherpesvirinae	Equid Herpesvirus 2	8	HQ247776, HQ247777, HQ247787, HQ247788, HQ247789, HQ247790, HQ247792, NC_001650

**TABLE A2:** Results of 25 runs of apcluster using established herpesvirus species trimmed to ~178 bp.

Cluster	Virus Species	Cluster	Virus Species
*Scenario 1 – Runs 1*, *4*, *5*, *6*, *10*, *12*, *14*, *16*, *17*, *18*, *19*, *20*, *21*, *23*, *24*		*Scenario 2 – Runs 3*, *9*, *11*, *15*	
1	Human Herpesvirus 1	1	Human Herpesvirus 1
2	Human Herpesvirus 2	2	Human Herpesvirus 2
3	Suid Herpesvirus 1	3	Suid Herpesvirus 1
4	Equid Herpesvirus 1, Equid Herpesvirus 9	4	Equid Herpesvirus 1, Equid Herpesvirus 9
5	Equid Herpesvirus 4	5	Equid Herpesvirus 4
6	Felid Herpesvirus 1	6	Felid Herpesvirus 1
7	Human Herpesvirus 3	7	Human Herpesvirus 3
8	Gallid Herpesvirus 2	8	Gallid Herpesvirus 2
9	Gallid Herpesvirus 3	9	Gallid Herpesvirus 3 (1 sequence)
10	Human Herpesvirus 4	10	Gallid Herpesvirus 3 (1 sequence)
11	Equid Herpesvirus 2	11	Human Herpesvirus 4
12	Human Herpesvirus 8	12	Equid Herpesvirus 2
13	Human Herpesvirus 5	13	Human Herpesvirus 8
14	Human Herpesvirus 6	14	Human Herpesvirus 5
15	Suid Herpesvirus 2	15	Human Herpesvirus 6
		16	Suid Herpesvirus 2
*Scenario 3 – Run 2*		*Scenario 4 – Run 7*	
1	Human Herpesvirus 1	1	Human Herpesvirus 1
2	Human Herpesvirus 2	2	Human Herpesvirus 2
3	Suid Herpesvirus 1 (1 Sequence)	3	Suid Herpesvirus 1
4	Suid Herpesvirus 1 (1 Sequence)	4	Equid Herpesvirus 1, Equid Herpesvirus 9
5	Equid Herpesvirus 1, Equid Herpesvirus 9	5	Equid Herpesvirus 4
6	Equid Herpesvirus 4	6	Felid Herpesvirus 1
7	Felid Herpesvirus 1	7	Human Herpesvirus 3
8	Human Herpesvirus 3	8	Gallid Herpesvirus 2 (1 sequence)
9	Gallid Herpesvirus 2	9	Gallid Herpesvirus 2 (1 sequence)
10	Gallid Herpesvirus 3	10	Gallid Herpesvirus 3 (1 sequence)
11	Human Herpesvirus 4	11	Gallid Herpesvirus 3 (1 sequence)
12	Equid Herpesvirus 2	12	Human Herpesvirus 4
13	Human Herpesvirus 8	13	Equid Herpesvirus 2
14	Human Herpesvirus 5	14	Human Herpesvirus 8
15	Human Herpesvirus 6	15	Human Herpesvirus 5
16	Suid Herpesvirus 2	16	Human Herpesvirus 6
		17	Suid Herpesvirus 2
*Scenario 5 – Run 8*		*Scenario 6 – Runs 13*, *25*	
1	Human Herpesvirus 1	1	Human Herpesvirus 1
2	Human Herpesvirus 2	2	Human Herpesvirus 2
3	Suid Herpesvirus 1 (1 Sequence)	3	Suid Herpesvirus 1
4	Suid Herpesvirus 1 (1 Sequence)	4	Equid Herpesvirus 1, Equid Herpesvirus 9
5	Equid Herpesvirus 1, Equid Herpesvirus 9	5	Equid Herpesvirus 4
6	Equid Herpesvirus 4	6	Felid Herpesvirus 1
7	Felid Herpesvirus 1 (1 Sequence)	7	Human Herpesvirus 3
8	Felid Herpesvirus 1 (1 Sequence)	8	Gallid Herpesvirus 2 (1 sequence)
9	Human Herpesvirus 3	9	Gallid Herpesvirus 2 (1 sequence)
10	Gallid Herpesvirus 2	10	Gallid Herpesvirus 3
11	Gallid Herpesvirus 3	11	Human Herpesvirus 4
12	Human Herpesvirus 4	12	Equid Herpesvirus 2
13	Equid Herpesvirus 2	13	Human Herpesvirus 8
14	Human Herpesvirus 8	14	Human Herpesvirus 5
15	Human Herpesvirus 5	15	Human Herpesvirus 6
16	Human Herpesvirus 6	16	Suid Herpesvirus 2
17	Suid Herpesvirus 2		
*Scenario 7 – Run 22*			
1	Human Herpesvirus 1		
2	Human Herpesvirus 2		
3	Suid Herpesvirus 1		
4	Equid Herpesvirus 1, Equid Herpesvirus 9		
5	Equid Herpesvirus 4		
6	Felid Herpesvirus 1		
7	Human Herpesvirus 3		
8	Gallid Herpesvirus 2, Gallid Herpesvirus 3		
9	Human Herpesvirus 4		
10	Equid Herpesvirus 2		
11	Human Herpesvirus 8		
12	Human Herpesvirus 5		
13	Human Herpesvirus 6		
14	Suid Herpesvirus 2		

*Note*: Results showed seven different sets of clusters, labeled here as Scenario 1 through Scenario 7. Because each cluster is considered to be a single OTU, this means that the 25 runs did not consistently cluster sequences into the exact same OTUs and instead did so in seven different ways. Each of the 25 runs that resulted in the same clustering scenario are listed, with Scenario 1 supported by the most number of runs (15 of the 25 runs clustered according to Scenario 1), and Scenarios 3, 4, 5, and 7 supported by only a single run. With the exception of equid Herpesviruses 1 and 2, Scenario 1 correctly clustered the sequences according to ICTV classifications. Although some scenarios resulted in the same number of clusters (e.g., Scenarios 2, 3, and 6), the composition of clusters (i.e. virus species in each cluster) is different for each scenario.

**TABLE A3:** Number of sequences required to accurately differentiate established herpesvirus species using affinity propagation.

Cluster	Sub‐family: Genus	Virus species
*1. Up to five sequences per species*		
1	Beta: *Cytomegalovirus*	HHV5: AF133619, MK867377, NC_006273, DQ180391, AB536875
2	Gamma: *Lymphocryptovirus*	HHV4: NC_007605, NC_009334
3	Gamma: *Percavirus*	Equid HV2: HQ247789, HQ247792, HQ247776, HQ247777, NC_001650
4	Gamma: *Rhadinovirus*	HHV8: AF005477, NC_009333
5	Alpha: *Simplexvirus*	HHV1: AB231458, AB231460, HQ123151, HQ123152, HQ123179
6	Alpha: *Simplexvirus*	HHV2: KF588405, HQ123175, JX905318, KM068901, KM068900
7	Alpha: *Varicellovirus*	Equid HV1: KF515985, KF434378, KF434382, KF434381, KC924817 Equid HV9: KX101094, NC_011644, KX101096
8	Alpha: *Varicellovirus*	Equid HV4: KF434385, KF434386, NC_001844, KF434387
9	Alpha: *Varicellovirus*	Felid HV1: AJ224971, NC_013590
10	Alpha: *Mardivirus*	Gallid HV2: L40431
11	Alpha: *Mardivirus*	Gallid HV2: NC_002229
12	Alpha: *Mardivirus*	Gallid HV3: MH939248, NC_002577
13	Alpha: *Varicellovirus*	HHV3: KU529537, AB059829, AB059830, AB059831, KU529522
14	Alpha: *Varicellovirus*	Suid HV1: KP279684
15	Alpha: *Varicellovirus*	Suid HV1: NC_006151
16	Beta: *Roseolovirus*	HHV6: AB283023, AB283024, NC_000898, NC_001664
17	Beta: unassigned	Suid HV2: AF268041, HQ686081, HQ113116, HQ686080, MG696113
2*. Up To three sequences per species*		
1	Beta: *Cytomegalovirus*	HHV5: NC_006273, DQ180391, AB536875
2	Gamma: *Lymphocryptovirus*	HHV4: NC_007605, NC_009334
3	Gamma: *Percavirus*	Equid HV2: HQ247792, HQ247776, HQ247777
4	Gamma: *Rhadinovirus*	HHV8: AF005477, NC_009333
5	Alpha: *Simplexvirus*	HHV1: HQ123152, HQ123151, HQ123179 HHV2: JX905318, KM068901, KM068900
6	Alpha: *Varicellovirus*	Equid HV1: KF434382, KF434381, KC924817 Equid HV9: KX101094, NC_011644, KX101096 Equid HV4: KF434386, NC_001844, KF434387
7	Alpha: *Varicellovirus*	Felid HV1: AJ224971, NC_013590
8	Alpha: *Mardivirus*	Gallid HV2: L40431, NC_002229 Gallid HV3: MH939248, NC_002577
9	Alpha: *Varicellovirus*	HHV3: AB059830, AB059831, KU529522
10	Alpha: *Varicellovirus*	Suid HV1: KP279684, NC_006151
11	Beta: *Roseolovirus*	HHV6: AB283024, NC_000898, NC_001664
12	Beta: unassigned	Suid HV2: HQ113116, HQ686080, MG696113
*3. Two sequences per species*		
1	Beta: *Cytomegalovirus*	HHV5: DQ180391, AB536875
2	Gamma: *Lymphocryptovirus*	HHV4: NC_007605, NC_009334
3	Gamma: *Percavirus*	Equid HV2: HQ247776, HQ247777
4	Gamma: *Rhadinovirus*	HHV8: AF005477, NC_009333
5	Alpha: *Simplexvirus; Varicellovirus*	HHV1: HQ123152, HQ123179 HHV2: KM068900, KM068901 Suid HV1: KP279684, NC_006151
6	Alpha: *Varicellovirus*	Equid HV1: KF434381, KC924817 Equid HV9: NC_011644, KX101096 Equid HV4: NC_001844, KF434387 Felid HV1: AJ224971, NC_013590
7	Alpha: *Mardivirus*	Gallid HV2: L40431, NC_002229
8	Alpha: *Mardivirus*	Gallid HV3: MH939248, NC_002577
9	Alpha: *Varicellovirus*	HHV3: AB059831, KU529522
10	Beta: *Roseolovirus*	HHV6: NC_000898, NC_001664
11	Beta: unassigned	Suid HV2: HQ686080, MG696113

*Note*: HHV stands for Human Herpesvirus and HV stands for Herpesvirus. When 2–5 sequences are available per species (i.e., “1. Up to Five Sequences Per Species”), discrepancies exist in clusters of some alphaherpesviruses when either PID is high (cluster 7, Equid HVs 1 and 9, see main text) or only two sequences represent the species (clusters 14 and 15, Suid HV1). When only 2–3 sequences are available per species (i.e., “2. Up to Three Sequences Per Species”), affinity propagation continues to over‐cluster (i.e. sequences representing different species are clustered together) alphaherpesviruses (e.g., clusters 5, 6, and 8) but can still accurately cluster at the level of virus genus. When only two sequences are included per species (i.e., “3. Two Sequences Per Species”), affinity propagation can no longer accurately cluster alphaherpesviruses at the genus level (e.g., cluster 5). Affinity propagation accurately clustered all included beta‐ and gammaherpesviruses, regardless of the number of sequences included per species.
